# Construction and Comprehensive Analysis of ceRNA Networks and Tumor-Infiltrating Immune Cells in Hepatocellular Carcinoma With Vascular Invasion

**DOI:** 10.3389/fbinf.2022.836981

**Published:** 2022-04-12

**Authors:** Shijiao Cai, Renle Du, Yuan Zhang, Zhengyi Yuan, Jie Shang, Yang Yang, Bin Han, Weilong Zhong, Hengjie Yuan, Zhengxiang Li

**Affiliations:** ^1^ Department of Pharmacy, Tianjin Medical University General Hospital, Tianjin, China; ^2^ Henan Institute of Medical and Pharmaceutical Sciences, Zhengzhou University, Zhengzhou, China; ^3^ Department of Gastroenterology and Hepatology, Tianjin Medical University General Hospital, Tianjin, China

**Keywords:** hepatocellular carcinoma, vascular invasion, competitive endogenous RNA network, immune infiltration, nomogram

## Abstract

**Background:** Hepatocellular carcinoma (HCC) is a common malignant cancer. Metastasis plays a critical role in tumor progression, and vascular invasion is considered one of the most crucial factors for HCC metastasis. However, comprehensive analysis focusing on competitive endogenous RNA (ceRNA) and immune infiltration in the vascular invasion of HCC is lacking.

**Methods:** The gene expression profiles of 321 samples, including 210 primary HCC cases and 111 HCC cases with vascular invasion, were downloaded from The Cancer Genome Atlas-Liver Hepatocellular Carcinoma project, and used in identifying significant differentially expressed lncRNAs (DElncRNAs), miRNAs (DEmiRNAs), and mRNAs (DEmRNAs). The RNAs associated with vascular invasion were used in constructing a ceRNA network. A multigene-based risk signature was constructed using the least absolute shrinkage and selection operator algorithm. We detected the fractions of 28 immune cell types in HCC through single-sample gene set enrichment analysis (ssGSEA). Finally, the relationship between the ceRNA network and immune cells was determined through correlation analysis and used in clarifying the potential mechanism involved in vascular invasion.

**Results:** Overall, 413 DElncRNAs, 27 DEmiRNAs, and 397 DEmRNAs were recognized in HCC. A specific ceRNA network based on the interaction among 3 lncRNA–miRNA pairs and 24 miRNA–mRNA pairs were established. A ceRNA-based prognostic signature was constructed and used in dividing samples into high- and low-risk subgroups. The signature showed significant efficacy; its 3- and 5-year areas under the receiver operating characteristic curves were 0.712 and 0.653, respectively. ceRNA and ssGSEA integration analysis demonstrated that PART1 (*p* = 0, R = −0.33) and CDK5R2 (*p* = 0.01, R = −0.15) were negatively correlated to natural killer cells.

**Conclusion:** This study demonstrated that vascular invasion in HCC might be related to PART1, and its role in regulating CDK5R2 and NK cells. A nomogram was developed to predict the prognosis of patients with HCC and demonstrated the value of the ceRNA network and tumor-infiltrating immune cells value in improving personalized management.

## Introduction

Hepatocellular carcinoma (HCC) is one of the most common malignant cancer types with poor prognosis and high incidence (Bray et al., 2018). HCC has poor prognosis partly because of its high recurrence rate after surgical operation, which can reach 10–20% at 5 years after liver transplantation (Duffy et al., 2007; Sotiropoulos et al., 2007) and 70% at 5 years after liver resection (Akoad and Pomfret, 2015). The presence of vascular invasion is a major adverse prognostic factor in HCC and is highly related to post–operative tumor recurrence and mortality (Jonas et al., 2001; Sumie et al., 2008; Rodriguez-Peralvarez et al., 2013). Known as the competitive endogenous RNA (ceRNA) network, the relationships among lncRNA, miRNA, and mRNA have been studied in many diseases (Zhang et al., 2020a; Zhang et al., 2020b; Han et al., 2021). However, the mechanism of the ceRNA network underlying vascular invasion in HCC remains unknown.

The enrichment scores of a pair of sample and gene set can be calculated through single-sample gene set enrichment analysis (ssGSEA) (Subramanian et al., 2005). The gene expression profile of a sample can be transformed into a gene set enrichment profile through ssGSEA. The enrichment scores of gene sets can represent the densities of tumor-infiltrating immune cells by defining immune cell-related gene sets. This transformation can better enable researchers to estimate tumor-infiltrating immune cells in the tumor microenvironment than flow cytometry and immunohistochemistry.

In this study, a ceRNA network was established based on the lncRNA, miRNA and mRNA expression profiles downloaded from The Cancer Genome Atlas database to identify the ceRNAs associated with vascular invasion. Immune cells were detected, and their proportions in HCC tumor tissues were estimated through ssGSEA. Additionally, a nomogram was developed to predict the prognosis of HCC with vascular invasion on the basis of significant ceRNAs. Finally, the relationship between vascular invasion-related ceRNA networks and immune cells was evaluated to clarify the potential mechanism involved in vascular invasion.

## Materials and Methods

### Data Collection and Differential Gene Expression Analysis

The data of 321 HCC patients cases, including 210 primary HCC cases and 111 HCC cases with vascular invasion, were downloaded from the TCGA-Liver Hepatocellular Carcinoma (LIHC) database. The “DESeq2” package in R software was used in finding differentially expressed long noncoding RNAs (DElncRNAs), miRNAs (DEmiRNAs) and mRNAs (DEmRNAs). The criteria were *p* < 0.05 and |log2 fold change (FC)| > 1. The “Pheatmap” and “ggplot2” packages in R software were used to draw the heat maps and volcano plots of the DERNAs, respectively.

### Construction of the ceRNA Network

First, interactions between lncRNAs and miRNAs were predicted using the starBase database (Li et al., 2014). Then, interactions between miRNAs and mRNAs were predicted using the “multiMiR” package. Finally, a ceRNA network was visualized using Cytoscape version 3.6.1 (Shannon et al., 2003).

### Functional Enrichment Analysis

The functions of 22 DEmRNAs in the ceRNA network were analyzed by Database for Annotation, Visualization, and Integrated Discovery (Huang da et al., 2009). The Entrez ID for each DEmRNA was obtained using the R package “org.Hs.eg.db.” The “clusterProfiler” and “ggplot2” packages were used to visualized the functions of the 22 DEmRNAs in terms of biological process (BP), cellular component (CC), and molecular function (MF).

### Construction of the Risk Score System

All the components of the ceRNA network associated with the prognosis of patients with HCC were analyzed through LASSO-penalized Cox regression. In addition, confounding factors were removed, and the number of genes was reduced. A Cox proportional hazards model was generated using the penalized maximum likelihood algorithm. Tenfold cross-validation was used in deriving the best lambda, minimizing the mean cross-validated error, and predicting the regression coefficients (*β*) of the multivariate Cox regression model. Finally, a prognosis risk score system based on three genes was generated. The formula used was as follows: risk score = β1 × exp1 + β2 × exp2 … + βn × expn. Herein, exp was the expression value of gene in the ceRNA network, and *β* was the regression coefficient of the multivariate Cox regression model (Lossos et al., 2004).

### Validation of the Prognostic ceRNA-Based Signature

Based on a previously established risk formula, each HCC sample gained a corresponding risk score. The median risk scores were set as the cutoff points, and all samples were classified into high- and low-risk clusters. Kaplan–Meier (K–M) curves were drawn using R package “survival” compared with log-rank tests. Time-dependent receiver operating characteristic (ROC) curves were used in validating the performance of the ceRNA-based signature in predicting prognosis.

### Establishment and Verification of the Nomogram

The regression coefficients in the multivariable Cox regression model were used in generating a nomogram. A nomogram plot that can integrate risk scores was used to predict 1, 3, and 5-year rates and develop a quantitative prognostic pool for patients with HCC. Then, a calibration curve that can present the predictive validity of the nomogram was plotted using the “rms” package.

### ssGSEA

Immune cell infiltration was determined using ssGSEA based on the integrated immune gene sets from published studies (Hanzelmann et al., 2013). The gene sets included 782 genes for predicting the abundance of 28 tumor-infiltrating immune cells (TIICs) in tissue samples. The proportions of 28 TIICs in the primary HCC and HCC with vascular invasion were estimated using ssGSEA. The Wilcoxon rank-sum test was used to identify significant immune cells between primary HCC and HCC with vascular invasion. Finally, the relationship between biomarkers and immune cells was explored through correlation analysis.

### Statistical Analysis

The Wilcoxon test was used in comparing two groups. Survival curves were analyzed using the K–M log rank test. Pearson analysis was used in computing the correlation coefficients. The results of ssGSEA with *p* < 0.05 were used in subsequent analysis. Two-tailed *p* < 0.05 was regarded as statistically significant. R software (version 3.6.3) was used in all statistical analyses.

## Results

### Identification of Significantly and Differentially Expressed Genes

**FIGURE 1:**
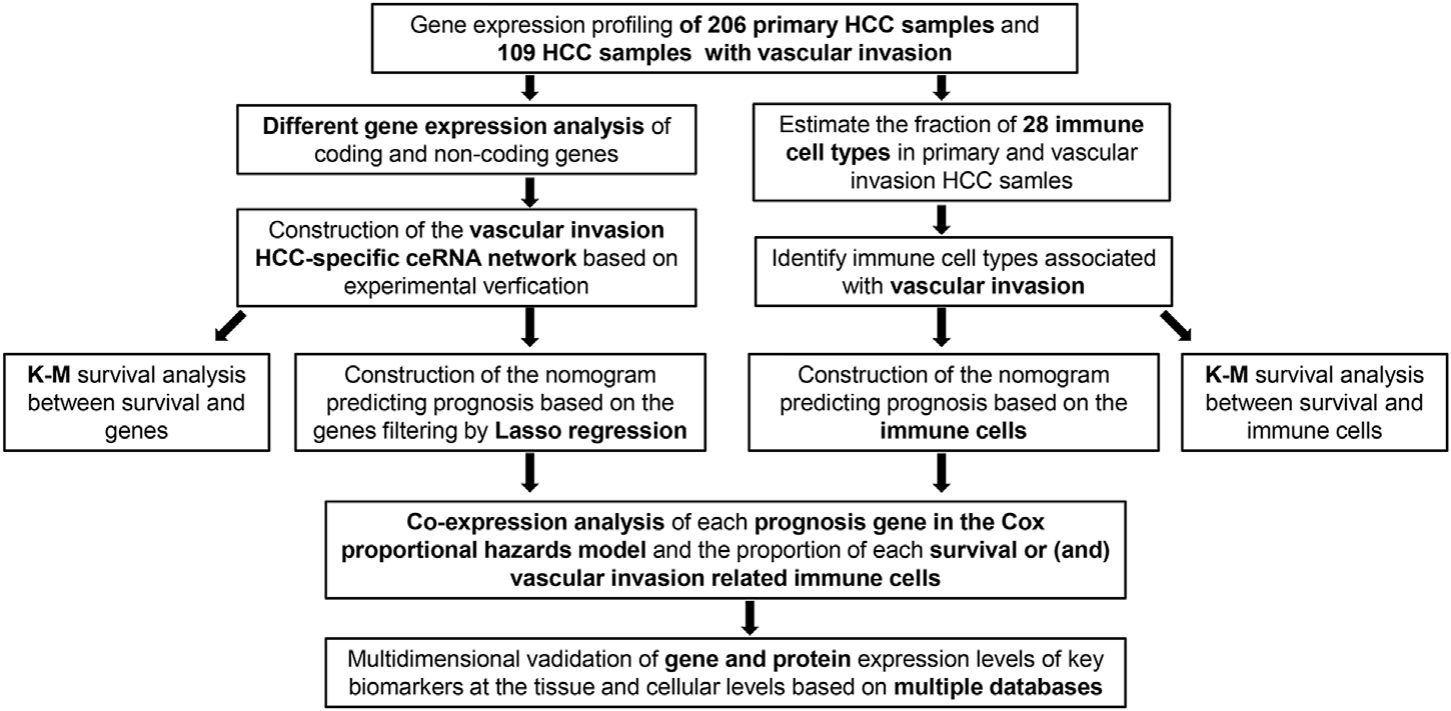
Flowchart of the analysis process.

**FIGURE 2:**
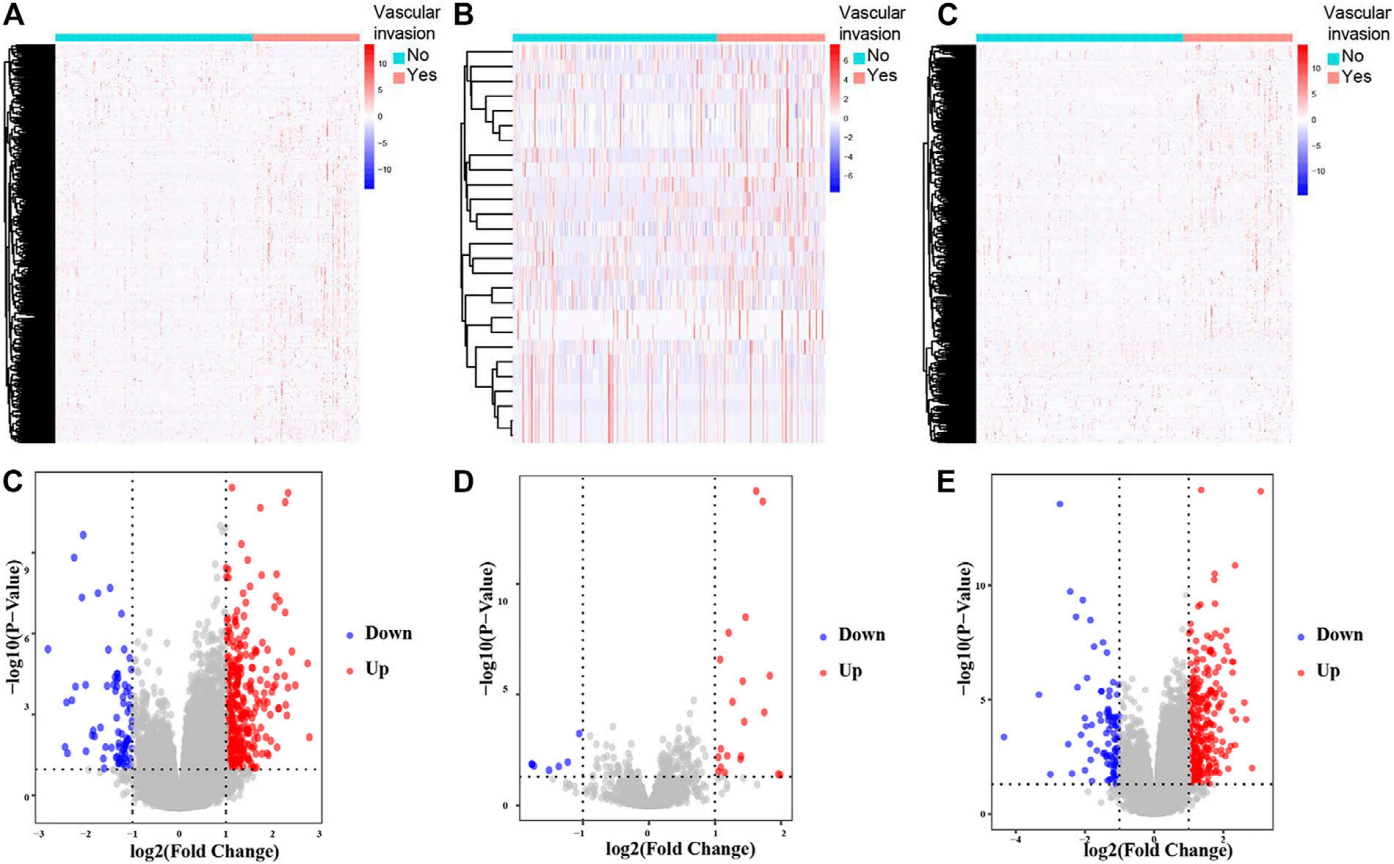
Hierarchical clustering heatmaps and volcano plots for all screened DElncRNAs, DEmiRNAs and DEmRNAs based on TCGA-LIHC data. Heatmaps located in the left panels represent DElncRNAs **(A)**, DEmiRNAs **(B)**, and DEmRNAs **(C)**. Annotations on top show the clustering of the samples. Green represents primary HCC; red represents HCC with vascular invasion. Volcano plots located in the right panels indicate DElncRNAs **(D)**, DEmiRNAs **(E)**, and DEmRNAs **(F)** with cutoff criteria of *p* < 0.05 and |FC| ≥ 1. Red, upregulated; blue, downregulated.

The schematic diagram of the analysis process of this study is illustrated in Figure 1. The criteria were *p* < 0.05 and |log2 FC| > 1. A total of 413 DElncRNAs (337 upregulated and 76 downregulated), 27 DEmiRNAs (20 upregulated and 7 downregulated), and 397 DEmRNAs (304 upregulated and 93 downregulated) between the 210 primary HCC and 111 HCC with vascular invasion obtained from the TCGA-LIHC database were found (Figures 2A–F).

### ceRNA Network Establishment and Survival Analysis

**FIGURE 3:**
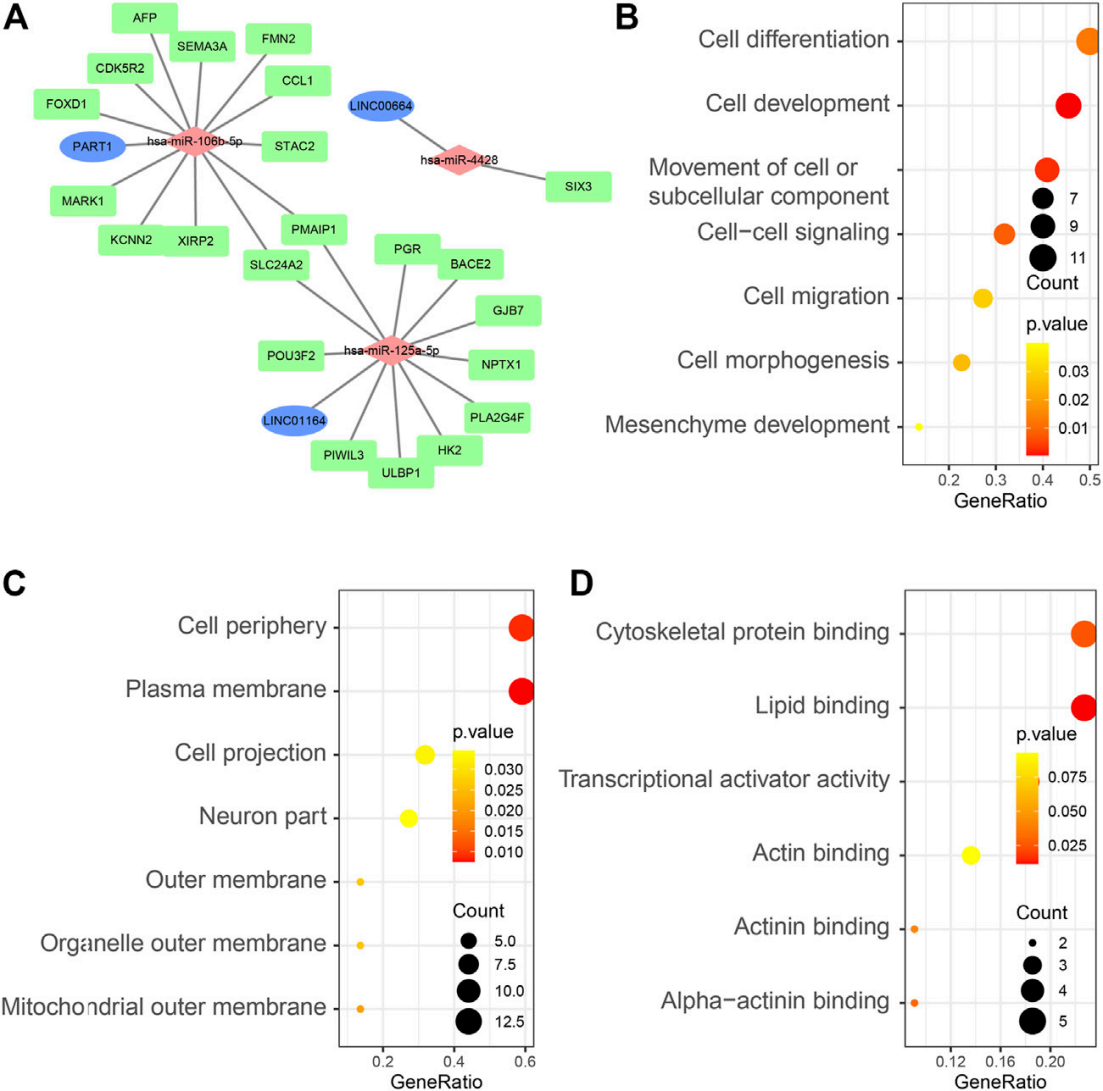
**(A)** Overview of the lncRNA–miRNA–mRNA ceRNA network of HCC with three lncRNA–miRNA pairs and 24 miRNA–mRNA pairs. Blue balls represent lncRNAs; red diamonds represent miRNAs and green rectangles represent mRNAs. GO enrichment analysis of 22 DEmRNAs in the ceRNA network: BP terms **(B)**, CC terms **(C)**, and MF terms **(D)**.

A ceRNA network was established and visualized on the basis of interactions among the three lncRNA–miRNA pairs and 24 miRNA–mRNA pairs (Figure 3A). Detailed information can be found in Supplementary Table S1. We performed gene ontology (GO) enrichment to explore the potential role of DEmRNAs in physiological processes. The result indicated that 22 DEmRNAs in the ceRNA network were primarily enriched during mesenchyme development, cell morphogenesis and cell migration in BP; plasma membrane in CC; and transcriptional activator activity in MF (Figures 3B–D).

**FIGURE 4:**
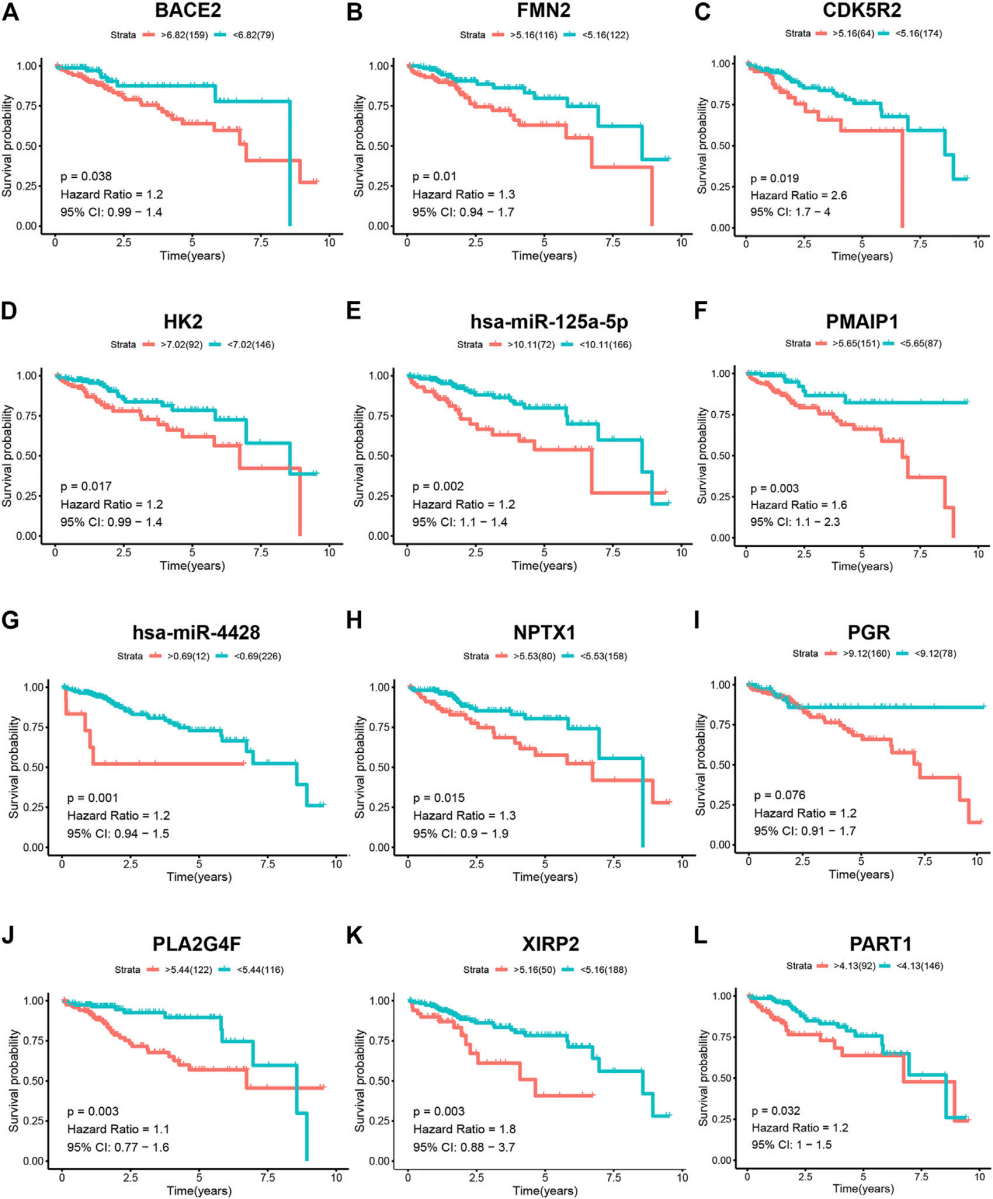
K–M survival analysis of the components of the ceRNA network in patients with HCC. K–M survival curves show that **(A)** BACE2 (*p* = 0.038), **(B)** FMN2 (*p* = 0.01), **(C)** CDK5R2 (*p* = 0.019), **(D)** HK2 (*p* = 0.017), **(E)** hsa-miR-125a-5p (*p* = 0.002), **(F)** PAMIP1 (*p* = 0.003), **(G)** hsa-miR-4428 (*p* = 0.001), **(H)** NPTX1 (*p* = 0.015), **(I)** PGR (*p* = 0.038), **(J)** PLA2G4F (*p* = 0.003), **(K)** XIRP2 (*p* = 0.003), and **(L)** PART1(*p* = 0.032) had significantly prognostic values.

A total of 261 patients with HCC with survival times greater than 30 days were selected for survival analysis. The prognosis of biomarkers involved in the ceRNA network related to vascular invasion in HCC was explored through K–M survival analysis. The results revealed that BACE2 (*p* = 0.038), FMN2 (*p* = 0.01), CDK5R2 (*p* = 0.019), HK2 (*p* = 0.017), hsa-miR-125a-5p (*p* = 0.002), PAMIP1 (*p* = 0.003), hsa-miR-4428 (*p* = 0.001), NPTX1 (*p* = 0.015), PGR (*p* = 0.038), PLA2G4F (*p* = 0.003), XIRP2 (*p* = 0.003), and PART1 (*p* = 0.032) showed significant difference (Figures 4A–L).

### Construction of Prognostic Risk Signature

**FIGURE 5:**
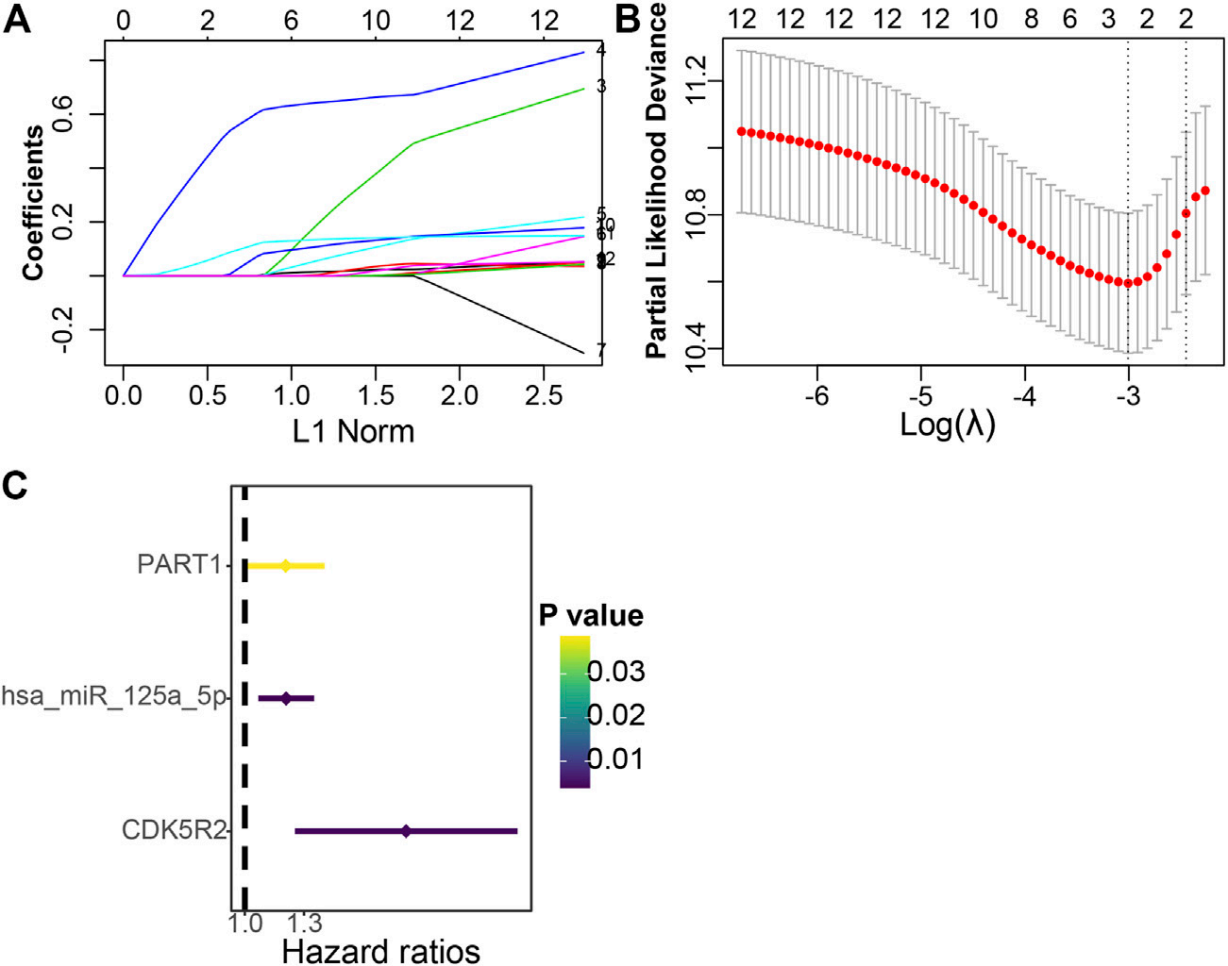
Establishment of the prognostic risk signature. **(A)** LASSO coefficient profiles of 12 candidate genes. A vertical line is drawn at the value selected through 10-fold cross-validation. **(B)** The vertical lines are plotted on the basis of the optimal data in accordance with the minimum criteria and 1-standard error criterion. The left vertical line represents the three genes finally identified. **(C)** Forest plots indicate the relationships of each gene subsets with OS. The unadjusted HRs are presented with 95% CIs.

A total of 12 ceRNA genes had significant prognosis. LASSO-penalized Cox regression and multivariate Cox regression analyses were performed, and three ceRNA genes (PART1, has-miR-125a-5p, and CDK5R2) associated with HCC prognosis were selected (Figures 5A–C). The three hub genes were incorporated into a risk score model, and the final risk score formula was as follows: risk score = (0.1818 × expression level of PART1) + (0.1837 × expression level of hsa-miR-125a-5p) + (0.7165 × expression level of CDK5R2). Finally, each HCC sample with a corresponding risk score was classified into low- and high-risk subgroups.

### Validation of Prognostic Risk Signature

**FIGURE 6:**
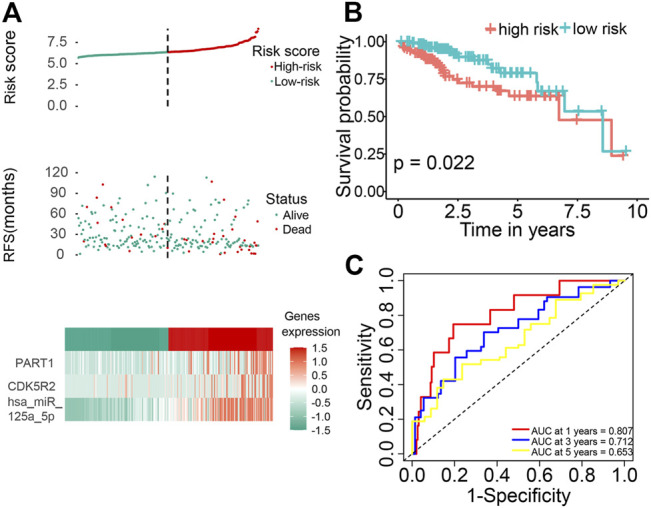
Identification of a three-ceRNA signature was significantly associated with OS of patients with HCC. **(A)** Risk score distribution, survival status, and three-ceRNA expression patterns of patients with HCC in the high-risk and low-risk groups. **(B)** The K–M curve of OS of the three-ceRNA signature. **(C)** Time-dependent ROC curves analysis at 1, 3, and 5 years.

The distributions of dot pot and risk score of survival status indicated that high-risk patients had shorter OS time (Figure 6A). The heatmap indicated the expression pattern of prognostic ceRNA between the high-risk group and low-risk groups (Figure 6A). The K–M survival curve demonstrated that the high-risk patients had significantly shorter OS times (Figure 6B; *p* = 0.022). In addition, we assessed the prognostic ability of the ceRNA signature by plotting the ROC curves at 1, 3 and 5 years. The results showed that the area under the curve (AUC) of the three ceRNA prognostic models was 0.807 (1 year), 0.712 (3 years) and 0.653 (5 years; Figure 6C).

### Construction of a Prognostic Nomogram

**FIGURE 7:**
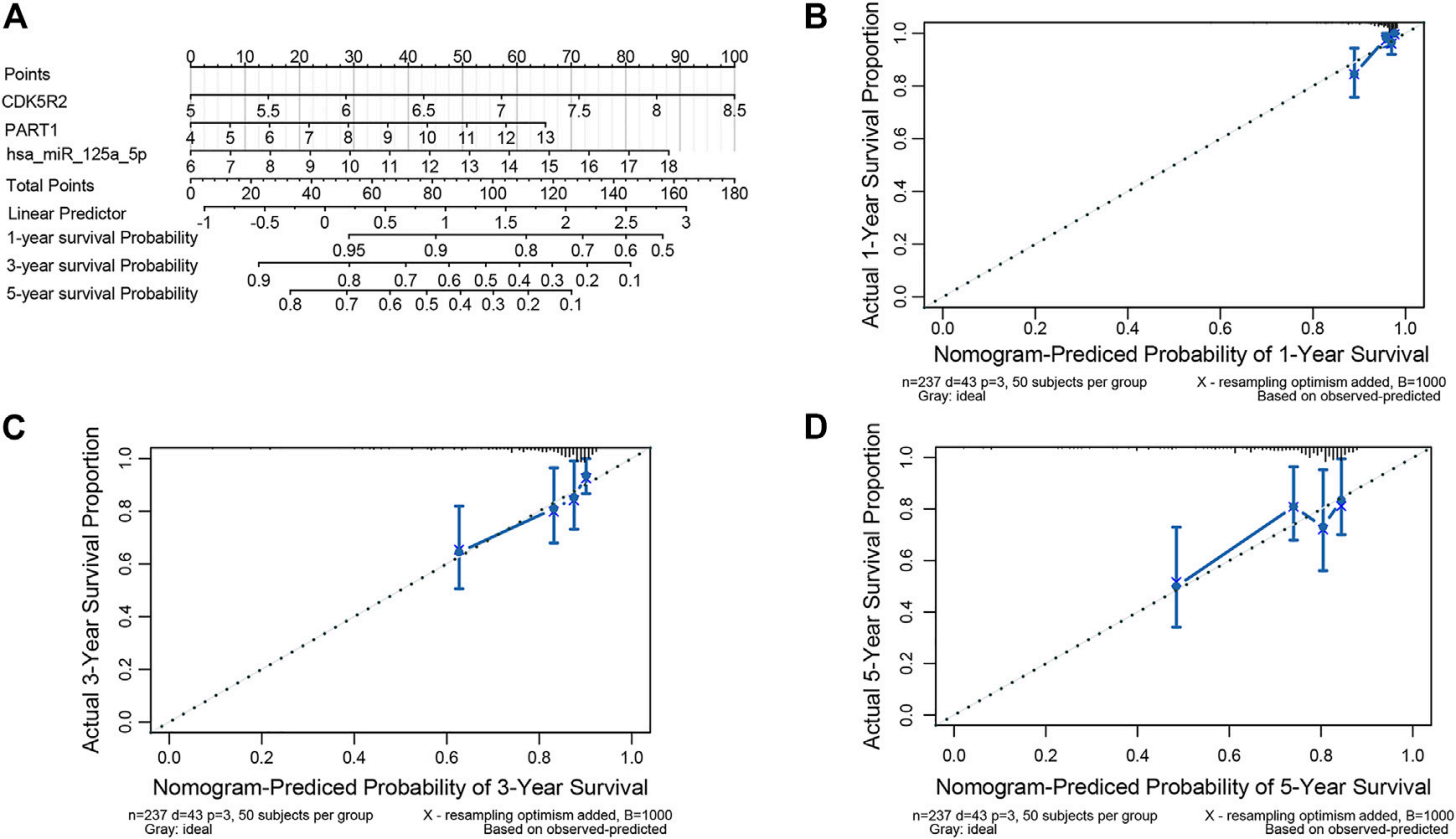
Nomogram for validating the prognostic efficiency of the risk signature. **(A)** The nomogram was assembled with a risk signature for predicting the survival of patients with HCC. The calibration plots of the nomogram for the probability of OS at 1 year **(B)**, 3 years **(C)**, and 5 years **(D)**.

We developed a prognostic nomogram consisting of three ceRNA components to provide a quantitative method that can predict the individual probability of survival in patients with HCC (Figure 7A). The bias-corrected lines of 1, 3 and 5 years in the calibration plot were close to the ideal curve (the 45° line), indicating the excellent predictive performance of the nomogram model (Figures 7B–D).

### Immune Cells Related to Vascular Invasion in HCC

**FIGURE 8:**
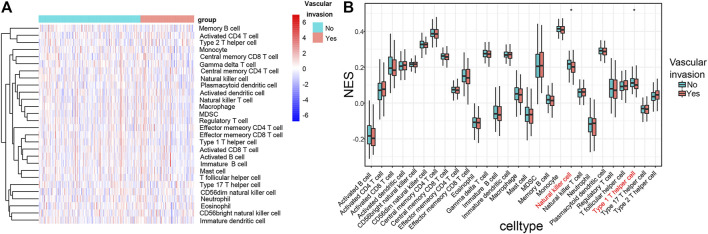
**(A)** Heat map of tumor-infiltrating immune cells in tumor tissues in patients with primary HCC and HCC with vascular invasion. Annotations on top show the clustering of the samples. Green represents primary HCC; red represents HCC with vascular invasion. **(B)** Bar plot for comparing the proportions of immune cells in primary HCC and HCC with vascular invasion. The proportions of NK cells (*p* < 0.05) and type 1 T helper cell (*p* < 0.05) in HCC with vascular invasion are relatively lower than those in the primary HCC with vascular invasion.

The composition of immune cells in HCC was evaluated using ssGSEA and visualized using a heat map (Figure 8A). The results of the Wilcoxon rank-sum test demonstrated that the proportions of natural killer (NK) cells (*p* < 0.05) and type 1 T helper cell (*p* < 0.05) in the HCC with vascular invasion were lower than those in primary HCC (Figure 8B). We applied ssGSEA to the GSE20017 dataset. The proportions of NK cells (*p* < 0.05) and type 1 T helper cell (*p* < 0.05) in HCC with vascular invasion were lower than those in primary HCC (Supplementary Figure S1).

### Comprehensive Analysis of Genes and Immune Cells

**FIGURE 9:**
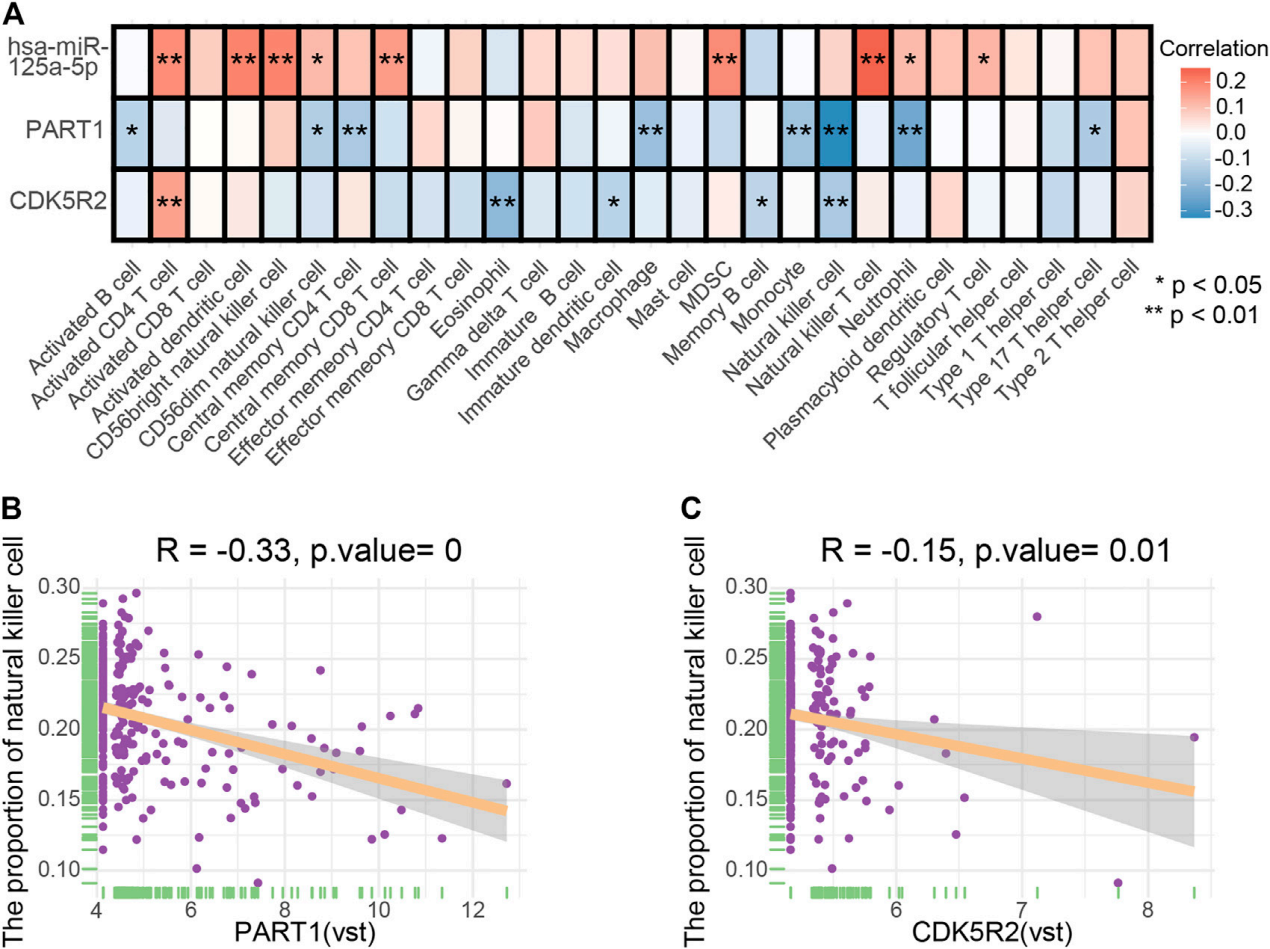
**(A)** Correlation relationships between three ceRNA and 28 tumor-infiltrating immune cells. **(B)** Scatter plots illustrate the correlation between PART1 and NK cells (*p* = 0, *R* = −0.33). **(C)** Scatter plots illustrate the correlation between CDK5R2 and NK cells (*p* = 0.01, *R* = −0.15).

The co-expression patterns of 28 immune cells were determined through correlation analysis (Supplementary Figure S2). In addition, the correlation between the expression of three ceRNA (PART1, has-miR-125a-5p and CDK5R2) and the infiltration levels of 28 immune cells was further analyzed and elucidated (Figure 9A). PART1 and the proportion of NK cells (Figure 9B, *p* = 0, *R* = −0.33), CDK5R2 and the proportion of NK cells (Figure 9C, *p* = 0.01, *R* = −0.15) were significantly and negatively correlated. The relationship between the expression of vascular invasion-specific ceRNAs and the proportion of NK cells in a different dataset was investigated using the GTBA database (http://guotosky.vip:13838/GTBA/). The results indicated that NK cell proportion had significantly negative correlations with PART1 (*p* = 2.61e−10, R = −0.321) and CDK5R2 expression (*p* = 0.0188, R = −0.122), which was consistent with our result (Supplementary Figure S3).

Dimensional validation using multiple online databases was performed to explore the expression of vascular invasion-specific ceRNAs (PART1 and CDK5R2) and the surface marker-coding genes of NK cells in different datasets. AKT serine/threonine kinase 3 (AKT3), C-X-C motif chemokine ligand 1 (CXCL1), follistatin-like 1 (FSTL1), leukocyte specific transcript 1 (LST1), fizzy and cell division cycle 20 related 1 (FZR1), and KN motif and ankyrin repeat domains 2 (KANK2) are the surface markers of NK cells (Charoentong et al., 2017). The Cancer Cell Line Encyclopedia (CCLE) reports the remarkable co-expression relationships between PART1 and the surface marker-coding genes of NK cells (Supplementary Table S2). Furthermore, we also analyzed the correlation between PART1 and immune markers in HCC from the TIMER database (Li et al., 2017) (Supplementary Figure S4). The correlation results are similar to those in CCLE. These findings suggest that PART1 is correlated with immune infiltration and may regulate NK cell polarization in HCC.

## Discussion

HCC is the common malignant cancer type with poor prognosis and high incidence. Histological tumor vascular invasion is considered as a crucial factor for HCC metastasis and/or recurrence (Roayaie et al., 2009). To date, the mechanisms of vascular invasion in HCC remains unclear. Thus, differentially expressed genes and TIICs in primary HCC and HCC with vascular invasion attracted our interest, which were rarely explored in previous studies.

In our study, we first determined differently expressed ceRNA between primary HCC and HCC with vascular invasion. Then, a nomogram was used as basis for predicting the outcomes of patients with HCC. The high AUC value and concordance index might contribute to the evaluation of vascular invasion. Based on the results of K–M survival analysis and correlation analysis, we inferred that the ceRNA regulatory mechanism of PART1 (lncRNA), hsa-miR-125a-5p (miRNA), CDK5R2 (mRNA), and NK cells plays crucial roles in the vascular invasion of HCC. Previous studies demonstrated that PART1 was highly expressed in HCC tissues and the PART1 expression profile was an effective tool for predicting early recurrence after surgical resection for HCC (Lv et al., 2018; Ye et al., 2019). Another study indicated that PART1 promoted the proliferation, migration and invasion of HCC cells by regulating the miR–149–5p/MAP2K1 axis (Zhou et al., 2020). The role of PART1 in HCC is consistent with our analysis.

The two commonly used computational methods for assessing the proportions of TIICs are ssGSEA and cell-type identification by estimating relative subsets of RNA transcripts (CIBERSORT) (Newman et al., 2015). In ssGSEA, marker genes were ranked by integrating differences among the empirical cumulative distributions of these genes based on their absolute expression levels in a single sample. Hence, this method was widely used in sample level enrichment analysis. It does not require data conversion when used in analyzing RNA sequencing data. The unnormalized RNA-seq count is a negative binomial distribution. By contrast, CIBERSORT requires input data that are Gaussian distributions. Therefore, RNA sequencing data must be converted into “microarray-like” data before analysis (Schelker et al., 2017). Furthermore, CIBERSORT can only estimate the proportion of 22 immune cell types, whereas ssGSEA can estimate 28 immune cell types. In the present study, we found different proportions of numerous immune cells in primary HCC and HCC with vascular invasion, and our results indicated that NK cells and type 1 T helper cells were closely related to vascular invasion.

NK cells are essential components of innate immunity against cancer, and changes in phenotype have been well studied in patients with HCC (Coulouarn et al., 2011). Moreover, available evidence has revealed that the frequency of circulating and intrahepatic NK cells was positively correlated with good prognosis in patients with HCC (Chew et al., 2012). In general, CD8^+^ T cells are important antitumor effector cells and their functions greatly depend on adequate CD4^+^ T cells (Gillgrass et al., 2014). Naive CD4^+^ T cells can be differentiated into various T helper (T_h_) cells, such as T_h_1, T_h_2, T_h_17, Tregs, and Tfh cells (Zhu et al., 2010). Previous studies have indicated that converting the unique T_h_2- to T_h_1-like profile in livers bearing metastatic tumors is a potential therapeutic approach for metastatic HCC (Budhu and Wang, 2006).

Correlation analysis indicated that NK cells were associated with PART1 (*p* = 0, *R* = −0.33) and CDK5R2(*p* = 0.01, *R* = −0.15). Therefore, we hypothesized that the interaction among PART1, hsa-miR-125a-5p, CDK5R2, and NK cells was highly relevant to vascular invasion in patients with HCC.

## Conclusion

Based on the ceRNA network, one nomogram was built and used in predicting survival and vascular invasion in patients with HCC. The nomogram was proven accurate by a high concordance index and AUC values. NK cells were downregulated in vascular invasion in patients with HCC and were associated with PART1 and CDK5R2. Furthermore, we inferred that the vascular invasion of HCC may depend on the interactions among PART1, hsa-miR-125a-5p, CDK5R2 and NK cells.

## Data Availability

The original contributions presented in the study are included in the article/Supplementary Material, further inquiries can be directed to the corresponding authors.

## Abbreviations

AUC, area under the curve; BP, biological processes; CC, cellular components; ceRNA, competitive endogenous RNA; CIBERSORT, cell-type identification by estimating relative subsets of RNA transcripts; DElncRNAs, differentially expressed long noncoding RNAs; DEmiRNAs, differentially expressed microRNAs; DEmRNAs, differentially expressed mRNAs; FC, fold change; GO, gene ontology; HCC, hepatocellular carcinoma; K–M, Kaplan–Meier; LASSO, least absolute shrinkage and selection operator; LIHC, liver hepatocellular carcinoma; MF, molecular function; NK, natural killer; OS, overall survival; ROC, receiver operating characteristic; ssGSEA, single-sample gene set enrichment analysis; TCGA-LIHC, The Cancer Genome Atlas Liver Hepatocellular Carcinoma; TIICs, tumor-infiltrating immune cells.
