# Supplementary material for: Construction and Comprehensive Analysis of ceRNA Networks and Tumor-Infiltrating Immune Cells in Hepatocellular Carcinoma With Vascular Invasion
Source: Front Bioinform. 2022 Apr 12;2:836981. doi: 10.3389/fbinf.2022.836981 (PMC9580849; doi:10.3389/fbinf.2022.836981)
Supplement: Supplementary file 1 [file Table1.pdf]

Supplementary Table1: Detailed information of the lncRNA–miRNA–mRNA ceRNA network in HCC.

**LncRNA**	**miRNA**	**Protein-coding RNA**
PART1	hsa-miR-106b-5p	CDK5R2
PART1	hsa-miR-106b-5p	AFP
PART1	hsa-miR-106b-5p	FOXD1
PART1	hsa-miR-106b-5p	SEMA3A
PART1	hsa-miR-106b-5p	FMN2
PART1	hsa-miR-106b-5p	CCL1
PART1	hsa-miR-106b-5p	STAC2
PART1	hsa-miR-106b-5p	PMAIP1
PART1	hsa-miR-106b-5p	SLC24A2
PART1	hsa-miR-106b-5p	MARK1
PART1	hsa-miR-106b-5p	KCNN2
PART1	hsa-miR-106b-5p	XIRP2
LINC01164	hsa-miR-125a-5p	PMAIP1
LINC01164	hsa-miR-125a-5p	SLC24A2
LINC01164	hsa-miR-125a-5p	PGR
LINC01164	hsa-miR-125a-5p	BACE2
LINC01164	hsa-miR-125a-5p	GJB7
LINC01164	hsa-miR-125a-5p	NPTX1
LINC01164	hsa-miR-125a-5p	PLA2G4F
LINC01164	hsa-miR-125a-5p	HK2
LINC01164	hsa-miR-125a-5p	ULBP1
LINC01164	hsa-miR-125a-5p	PIWIL3
LINC01164	hsa-miR-125a-5p	POU3F2
LINC00664	hsa-miR-4428	SIX3
