# Supplementary material for: Construction and Comprehensive Analysis of ceRNA Networks and Tumor-Infiltrating Immune Cells in Hepatocellular Carcinoma With Vascular Invasion
Source: Front Bioinform. 2022 Apr 12;2:836981. doi: 10.3389/fbinf.2022.836981 (PMC9580849; doi:10.3389/fbinf.2022.836981)
Supplement: Supplementary file 2 [file Table2.pdf]

Supplementary Table2: Correlation between PART1 and the surface markers of NK cells for HCC in Cancer Cell Line Encyclopedia.

	**Gene marker**	**PART1**	
		**R**	**P**
**NK cell**	AKT3	-0.382	0.0598
	CXCL1	-0.435	*
	FSTL1	-0.398	*
	LST1	-0.505	**
	FZR1	-0.482	*
	KANK2	-0.566	**
