# Supplementary material for: Construction and Comprehensive Analysis of ceRNA Networks and Tumor-Infiltrating Immune Cells in Hepatocellular Carcinoma With Vascular Invasion
Source: Front Bioinform. 2022 Apr 12;2:836981. doi: 10.3389/fbinf.2022.836981 (PMC9580849; doi:10.3389/fbinf.2022.836981)
Supplement: Supplementary file 3 [file Image4.pdf]

Supplementary Figure4

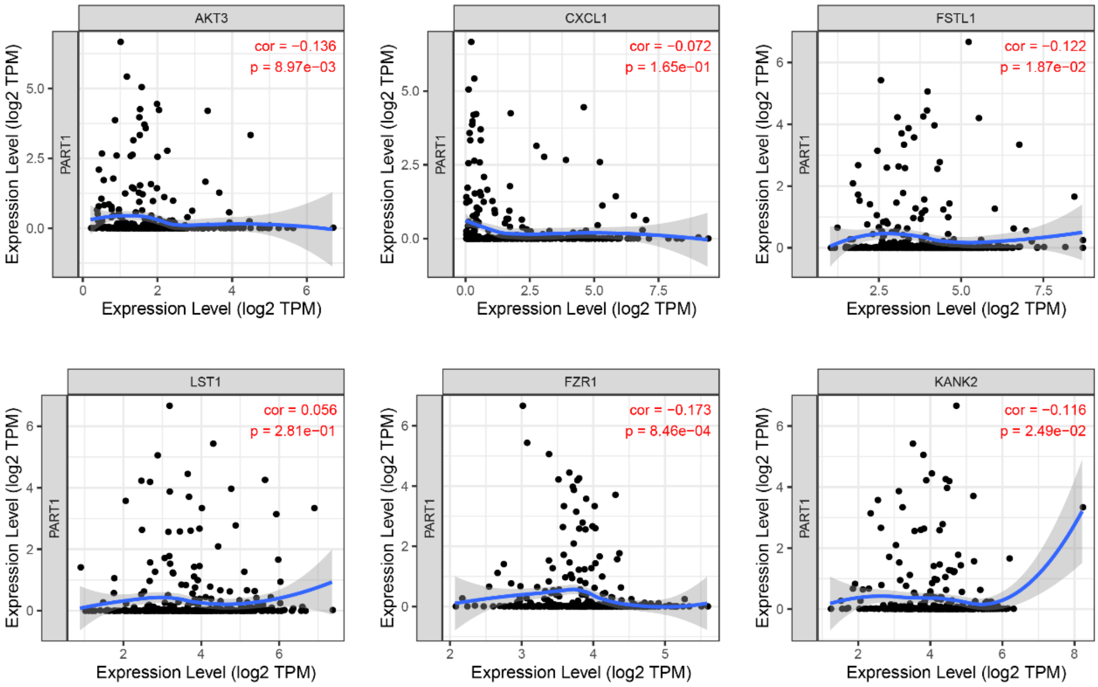

Correlations between PART1 and the surface markers of NK cells (AKT3, CXCL1, FSTL1, LST1, FZR1, and KANK2) for HCC in TIMER.
