# Supplementary figures and images for: Construction and Comprehensive Analysis of ceRNA Networks and Tumor-Infiltrating Immune Cells in Hepatocellular Carcinoma With Vascular Invasion
Source: Front Bioinform. 2022 Apr 12;2:836981. doi: 10.3389/fbinf.2022.836981 (PMC9580849; doi:10.3389/fbinf.2022.836981)

## Supplementary Figure2

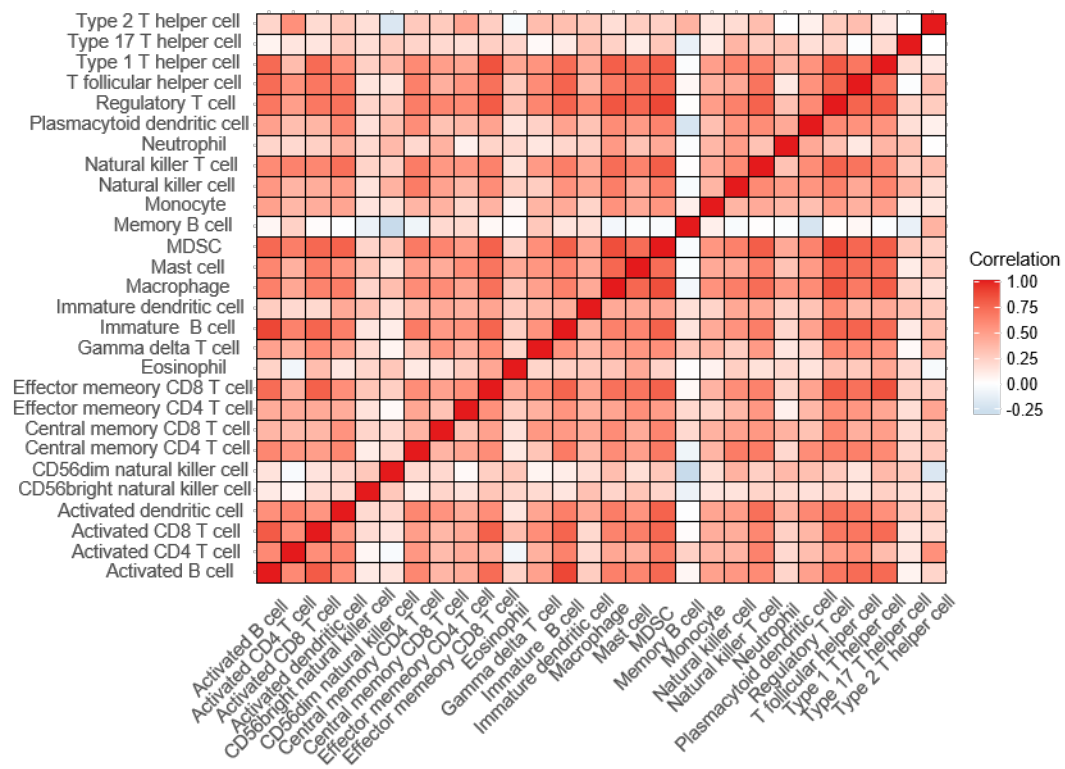

Correlation analysis of 28 tumor-infiltrating cells.

Supplement: Supplementary file 4 [file Image2.pdf]
