# Supplementary material for: Construction and Comprehensive Analysis of ceRNA Networks and Tumor-Infiltrating Immune Cells in Hepatocellular Carcinoma With Vascular Invasion
Source: Front Bioinform. 2022 Apr 12;2:836981. doi: 10.3389/fbinf.2022.836981 (PMC9580849; doi:10.3389/fbinf.2022.836981)
Supplement: Supplementary file 5 [file Image3.pdf]

Supplementary Figure 3

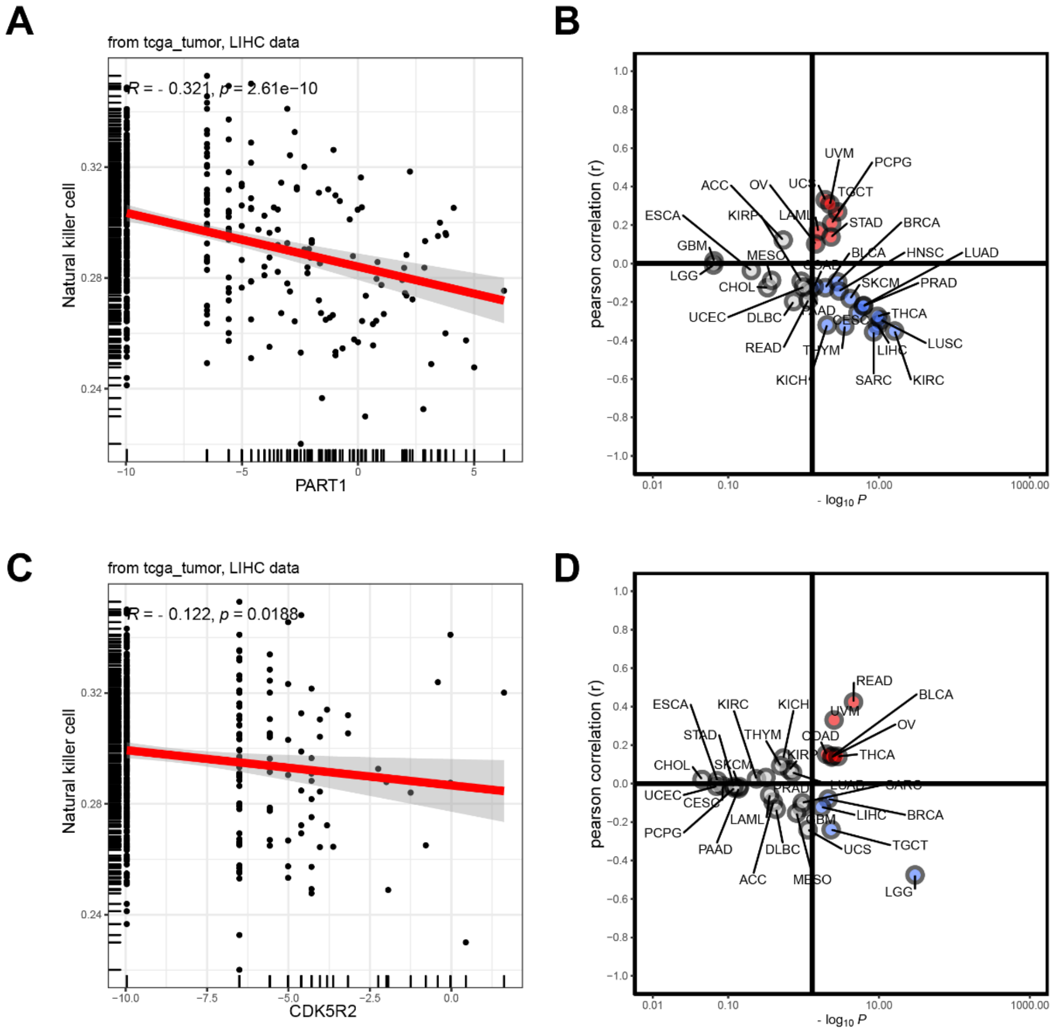

Relationship of PART1 and CDK5R2 expression with the proportion of NK cells in the GTBA database. **(A)** Scatter plots illustrate the correlation between PART1 and NK cells (*P* = 2.6e–10, *R* = –0.321). **(B)** Circle diagram illustrate the correlation between PART1 and NK cells in pan-cancer. Red, positive correlation; blue, negative correlation. **(C)** Scatter plots illustrate the correlation between CDK5R2 and NK cells (*P* = 0.0188, *R* = –0.122). **(D)** Circle diagram illustrate the correlation between CDK5R2 and NK cells in pan-cancer. Red, positive correlation; blue, negative correlation.
