# Supplementary material for: Construction and Comprehensive Analysis of ceRNA Networks and Tumor-Infiltrating Immune Cells in Hepatocellular Carcinoma With Vascular Invasion
Source: Front Bioinform. 2022 Apr 12;2:836981. doi: 10.3389/fbinf.2022.836981 (PMC9580849; doi:10.3389/fbinf.2022.836981)
Supplement: Supplementary file 6 [file Image1.pdf]

Supplementary Figure1

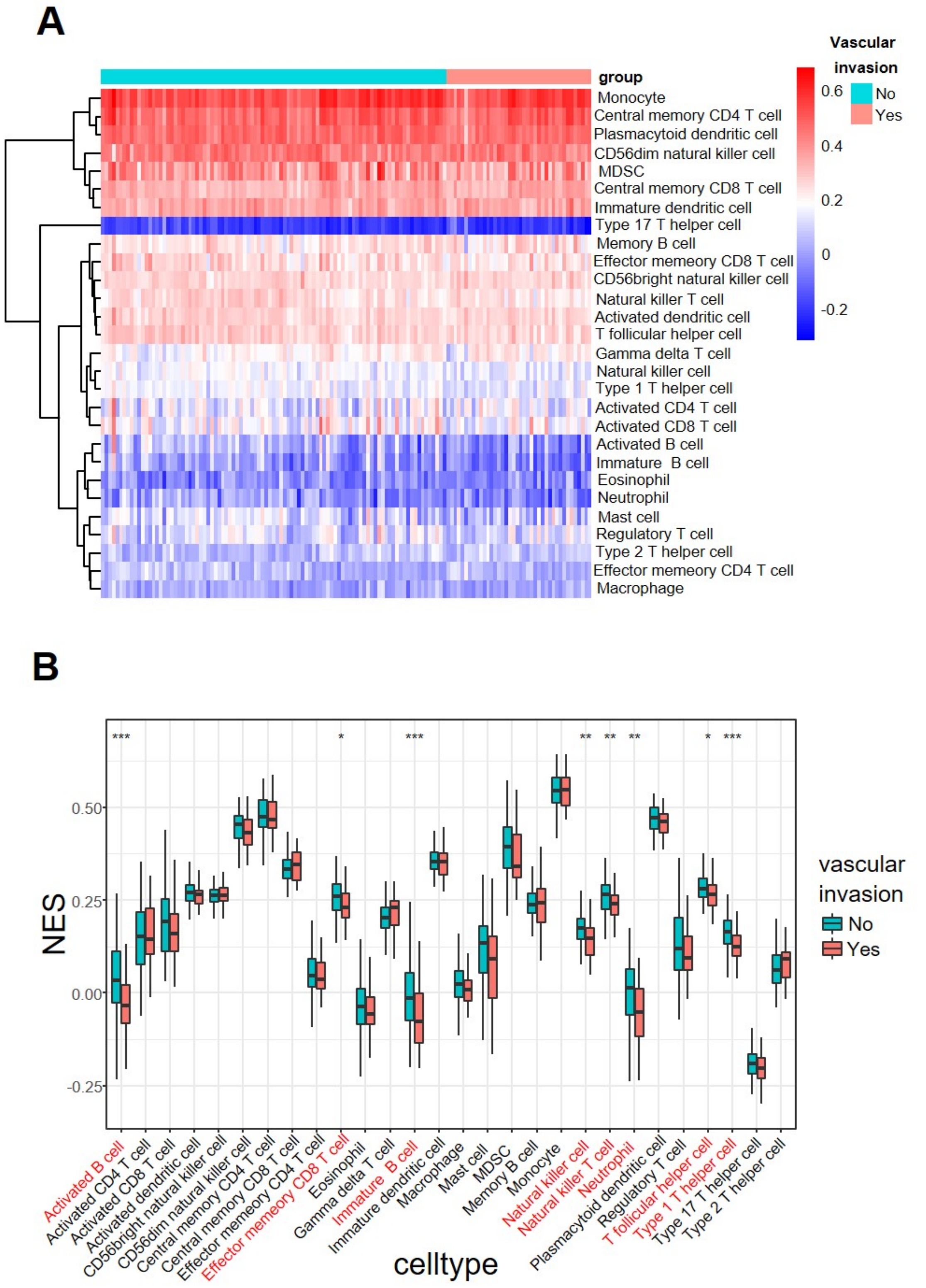

(A) Heat map of tumor-infiltrating cells in tumor tissues in patients with primary HCC and the HCC with vascular invasion in the GSE20017 dataset. Annotations on top show the clustering of samples. Green represents primary HCC, red represents HCC with

vascular invasion. (B) Bar plot for comparing the proportions of immune cells of HCC and HCC with vascular invasion.
